# Systemic lupus erythematosus presenting as lupus erythematosus tumidus and lupus nephritis: a case report

**DOI:** 10.1186/s13256-023-03981-3

**Published:** 2023-06-14

**Authors:** Meriam Hajji, Imen Gorsane, Samaraa Badrouchi, Noureddine Litaiem, Soumaya Rammeh, Fethi Ben Hamida, Ezzeddine Abderrahim

**Affiliations:** 1 Department of Medicine A, Charles Nicolle Hospital, Tunis, Tunisia; 2 Kidney Pathology Laboratory LR00SP01, Charles Nicolle Hospital, Tunis, Tunisia; 3 Department of Dermatology, Charles Nicolle Hospital, Tunis, Tunisia; 4 Department of Pathology, Charles Nicolle Hospital, Tunis, Tunisia; 5 Faculty of Medicine of Tunis, El Manar University, Tunis, Tunisia

**Keywords:** Lupus erythematosus tumidus, Lupus nephritis, Glomerulonephritis, Tumid lupus, SLE, Systemic lupus

## Abstract

**Background:**

Lupus nephritis and lupus erythematosus tumidus (LET) are uncommon manifestations of systemic lupus erythematosus (SLE), and their coexistence as the initial presentation of SLE is exceedingly rare. Here, we report such a case, emphasizing the diagnostic challenges and therapeutic implications of this unusual association.

**Case report:**

A 38-year-old North African woman presented in Nephrology department with a history of lower extremity edema, fatigue, and weight loss of 3 kg in 4 weeks. Physical examination revealed LET lesions on the chest and the Neck. Laboratory investigations showed lymphopenia, low C3 and C4 complement levels, positive antinuclear antibodies, anti-dsDNA antibodies, and anti-SSA/Ro antibodies. Renal function tests showed normal serum creatinine and nephrotic proteinuria. Renal biopsy revealed Class V lupus nephritis. Skin biopsy confirmed the diagnosis of LET, with the presence of lymphohistiocytic infiltrates and dermal mucin. The patient was diagnosed with SLE based on the 2019 EULAR/ACR criteria and treated with prednisone (1 mg/kg/day) and hydroxychloroquine. She showed significant improvement in her cutaneous and renal symptoms at 6 and 12 months follow-up.

**Conclusion:**

The rarity of the coexistence of LET and lupus nephritis as the initial manifestation of SLE, especially in the North African population, underscores the need for further research to elucidate the immunopathogenic mechanisms and prognostic factors associated with this association.

## Introduction

Systemic lupus erythematosus (SLE) is a multisystem autoimmune disorder that can present with diverse clinical manifestations, including cutaneous involvement and renal disease. However, lupus erythematosus tumidus (LET), a rare subtype of cutaneous lupus, is an unusual initial presentation of SLE, and lupus nephritis as the sole or predominant feature at onset is also infrequent.

Therefore, we present a remarkable case of a patient who was diagnosed with SLE based on the concurrent presence of membranous lupus nephritis and LET, without overt systemic symptoms.

## Case report

A 38-year-old North African female patient was referred to Nephrology department with lower extremity edema for 1 month. She had a history of Primary hypothyroidism and secondary infertility with amenorrhea for a year. She reported asthenia and a weight loss of 3 kg in 1 month. She was apyretic. She had no arthralgias or myalgias. She did not suffer from oral or ocular dryness She did, instead, report the appearance of skin patches, which were photosensitive and showed fine scaling and associated pruritus.

**Fig. 1:**
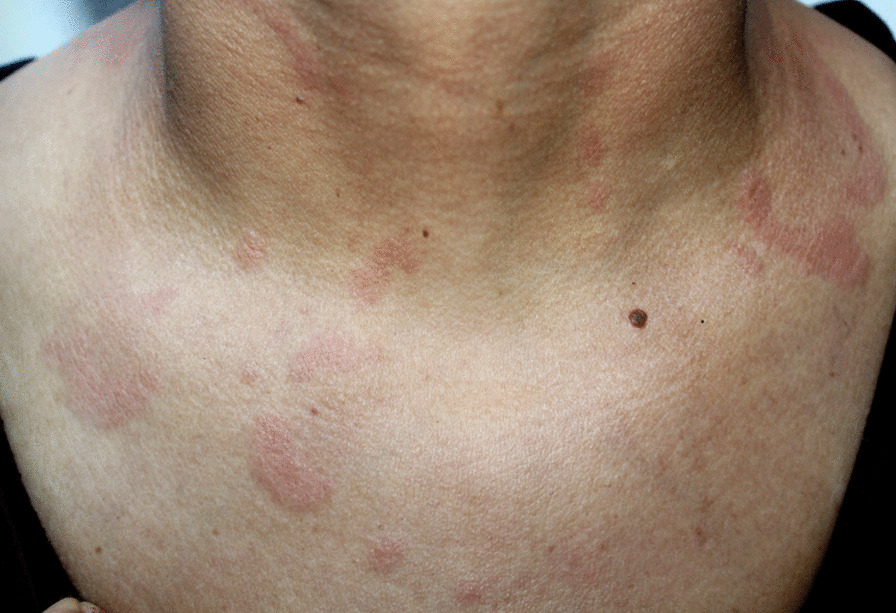
Lupus tumidus lesions on our patient’s Chest: Erythematous, firm, and asymptomatic plaques

**Fig. 2:**
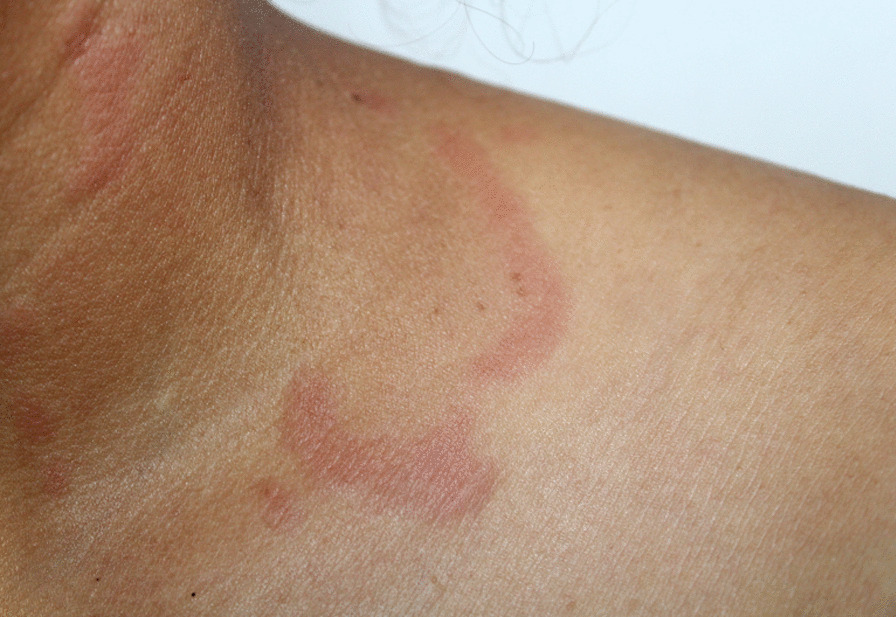
Lupus tumidus lesions on the patient’s neck: Annular or polycyclic, with a central clearing and a raised border

On examination, her blood pressure was 110/70 mmHg, pulse was 99/min, temperature was 36.3 °C, and respiratory rate was 18/min. Urinalysis showed 2 + proteinuria and 1 + hematuria. The skin examination revealed firm curved erythematous patches on the neck, face, and chest and moderate edema of both lower limbs. There was no atrophy, scarring or dyspigmentation over the skin lesion (Figs. 1 and 2).

**Fig. 3:**
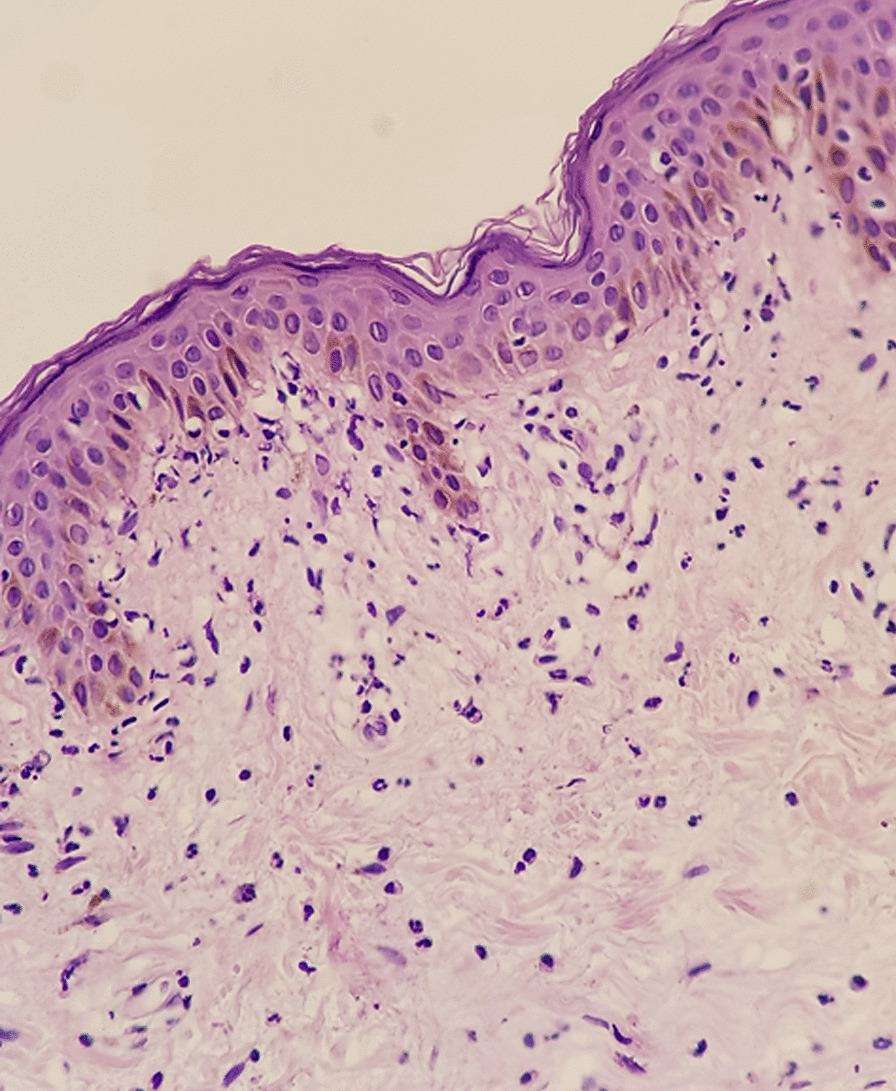
Hematoxylin–eosin: atrophy of the spinous layer, numeric and volumetric reduction of interpapillary ridges in the epidermis. Moderate perivascular infiltrate in the dermis

Initial laboratory investigations showed a decreased serum albumin level of 33.5 g/L then 28.2 g/l (normal rage: 35–50 g/L) and proteinuria of 4.5 g/day (urinary total volume 1000 mL). Microscopic examination of the urine showed microscopic hematuria (80–100 cells/HPF) and leukocyturia (50,000/mL). Electrophoresis of serum proteins showed a polyclonal peak in gamma globulins at 33.6 g/L. Serum creatinine was 58 μmol/L. The patient's blood test results showed a leukocyte count of 4900/mm3, with 1100/mm^3^ being lymphocytes, hemoglobin level of 12 g/dL, and a platelet count of 178,000/mm^3^. The patient's antibody test showed a positive result with a titer of 1:1800 and homogenous pattern. Additionally, the patient tested positive for anti-double-stranded DNA antibodies with a titer of 1:40, and for anti-Sm (+ +), anti-RNP (+ +), and anti-SSA antibodies (+). The complement C3 and C4 levels were reduced to 42 mg/L (normal range: 81–157 mg/dL) and 7 mg/dL (normal range: 13–39 mg/dL), respectively, while CH50 was 9.5 U/mL (normal range: 31.7–95 U/mL). The results for Anti-glomerular basement membrane antibody, anti-neutrophil cytoplasmic antibody, Coombs' test, antiphospholipid antibody, serum cryoglobulins, serum and urine immunofixation electrophoresis, and anti-PLA2R antibody were all negative. The skin biopsy confirmed the diagnosis of LET, with the presence of inflammatory lymphohistiocytic infiltrates and dermal mucin on and below the skin surface, with little or no involvement of the epidermis or dermo-epidermal layer (Fig. 3). Direct immunofluorescence of skin biopsy confirmed LET by the presence of CD3/CD4 lymphocytes.

Ultrasound examination of the kidneys showed normal-sized kidneys (left 11.8 cm; right 11.7 cm). A renal biopsy was performed. Light microscopy showed that 8/34 glomeruli were sclerotic and the rest showed segmental mesangial deposits and infiltration of neutrophils, but no mesangial cell or stromal proliferation. The glomerular basement membrane was diffusely thickened with segmental spikes. The renal interstitium contained some inflammatory infiltrates, and the tubules showed small focal atrophy and scattered proteinaceous and red blood cell casts. There was intimal fibrous proliferation and sclerosis of small arteries. Congo red stain for amyloid was negative. Direct immunofluorescence showed full-house staining along the mesangium and the capillary loops with subepithelial immune deposits of IgG (3 +), IgA (+), IgM (1 +), C3 (2 +), C1q (1 +), fibrinogen (2 +), albumin (−), kappa (2 +), lambda (3 +).

The patient met six of the EULAR/ACR 2019 classification criteria for systemic lupus erythematosus (SLE) [1], with a total score of 31 including glomerular proteinuria, and a Class V renal biopsy. Positive ANA results, positive anti-dsDNA and anti-Sm antibodies and reduced complement C3 and C4 proteins. Symptomatic treatment was based on anti ptoteinuric treatment with ACE inhibitors (Captopril _25_ 1 table × 2/day) and substantive treatment combined oral corticotherapy at 1 mg/kg/day (prednisone _5_ 12 tablets/day) for 1 month followed by tapering 5 mg/15 days and hydroxychloroquine _200_ twice a day. No DMARD therapy for SLE has been initiated. After 1 month, the skin lesions regressed. At 6 and 12 months follow up, proteinuria improved to respecively 0.8 g/24 hour and 0.5 g/24 hour.

At the latest check-up, 22 months after the initial presentation, the patient’s proteinuria was 684 mg/24 hour, serum creatinine was 51 µmol/l, and there was complete resolution of the lesions.

## Discussion

Lupus erythematosus tumidus (LET) is a chronic skin inflammatory disease that was first reported in 1909 [2]. The coexistence of SLE and LET in the same patient, is extremely rare, seen only in 2–15% of cases in a retrospective case study [3]. The skin lesions of LET can be divided into two categories: those specific to systemic lupus erythematosus (SLE) and those that are not [4]. The latter are considered a significant indicator of disease progression to the systemic form of lupus [5]. LET primarily affects sun-exposed areas and presents as erythematous, edematous, succulent plaques that are non-scarring [6, 7]. LET rash as seen in our patient, was usually highly photosensitive. Also usually there is no atrophy, scarring or dyspigmentation which is typically seen in other forms of chronic cutaneous lupus [8–10].

Some researchers consider LET to be a separate form of chronic cutaneous lupus erythematosus (CLE) given that, the incidence of LET is estimated to be 16% when considering all forms of cutaneous lupus [11]. The histopathological features of chronic cutaneous lupus erythematosus (CLE) are characterized by perivascular lymphocytic infiltration and interstitial mucin deposition [12], with a notable absence of the typical features of acute CLE, such as interface dermatitis, epidermal involvement, and hair follicle alteration. In comparison to discoid lupus erythematosus, CLE tends to exhibit a more prominent mucin deposition, as observed in previous studies [7, 9, 13].

The progression to SLE has been reported in 12–18% of cases [11]. The relationship between SLE and LET is not fully understood and there have only been a few reported cases, primarily in Europe [9–12]. In a study of 40 patients diagnosed with LET, none of them showed evidence of systemic involvement or met the criteria for a diagnosis of SLE [8].

Our case is also unique because the patient is from North Africa, whereas most of the reported cases and series are from Europe, Asia, and America, and in relation to immunologic aspects, in majority of LET patients, anti-nuclear antibody (ANA) is negative, ANA positivity is seen in less than 20% of patients [14–16]. The positivity of other commonly systemic lupus associated serologies such as dsDNA, SSA and SSB or low C3 and C4 complement levels are rarely seen in LET [17], making this case unique due to their presence. Anti-SSA and, sometimes, anti-SSB antibodies have been associated with photosensitivity in subacute cutaneous lupus erythematosus, but this has not been seen in patients with LET [15–17]. In one Japanese series of ten patients with LET, four patients had an ANA titer of 1:160, four tested positive for anti-Ro/SS-A, and two tested positive for anti-La/SS-B [18]. Although systemic symptoms are not commonly observed in patients with LET, one case was reported to have developed systemic symptoms during the course of the disease, leading to a reconsideration of the diagnosis as early-stage SLE. LET has also been reported to occur during the course of Systemic Sclerosis [19].

Our case is as well significant because of the rare association between LET and severe SLE with lupus nephritis (NL). In our patient, the CH 50, C3 and C4 fractions of complement were lowered by consumption after the activation of the classical pathway observed in SLE, in particular with renal involvement.

Hydroxychloroquine sulfate has been shown to be effective in treating LET, with a daily dose of 6–6.5 mg/kg [20, 21]. Systemic corticosteroids or immunosuppressants may not be necessary, but were prescribed for our patient due to a concurrent diagnosis of lupus nephritis. Photoprotection is also important as LET is highly photosensitive [22].

This case underscores the importance of considering SLE in the differential diagnosis of patients with atypical skin lesions or unexplained renal dysfunction, even in the absence of classic signs or symptoms. Moreover, the coexistence of LET and lupus nephritis may reflect distinct immunopathogenic mechanisms and prognostic implications, warranting further investigation.

## Conclusion

The uncommon occurrence of lupus erythematosus tumidus (LET) as an initial presentation of systemic lupus erythematosus (SLE), especially in conjunction with severe lupus nephritis, in our patient highlights the diagnostic challenges and clinical heterogeneity of SLE. Our report contributes to the expanding knowledge of the clinical spectrum of SLE, and emphasizes the need for a multidisciplinary approach in the management of these complex cases.

## Data Availability

Not applicable.
